# Anxiety and Depression in Mild and Moderate COPD Patients: An Observational, Cross-Sectional Study in Greece

**DOI:** 10.3390/diseases13080266

**Published:** 2025-08-17

**Authors:** Effimia Kamariotou, Diamantis Chloros, Dionisios Spyratos, Dionisia Michalopoulou, Ioanna Tsiouprou, Lazaros Sichletidis

**Affiliations:** 1 National Health System Pulmonary Department (NHS), Pulmonary Department, G. Papanikolaou Hospital, Exochi, 57010 Thessaloniki, Greece; efi_kamariwtou@hotmail.com; 2 Pulmonary Department, Aristotle University of Thessaloniki, G. Papanikolaou Hospital, Exochi, 57010 Thessaloniki, Greece; diospyrato@yahoo.gr (D.S.); d.michalopoulou@thessaloniki.gr (D.M.); joanna_tsi@hotmail.com (I.T.); sichlet@auth.gr (L.S.)

**Keywords:** depression, anxiety, mood disorders, chronic obstructive pulmonary disease, hospital anxiety and depression scale questionnaire

## Abstract

Background: In this study, we investigated patients in the early stages of COPD to support the hypothesis that symptoms of anxiety and depression are related to mild and moderate COPD and not only to the chronic complications that accompany severe disease. Methods: A total of 250 mild to moderate COPD patients were randomly selected from a population of 5239 individuals who were part of a study on early COPD detection and smoking cessation that was carried out in Central Macedonia, Greece. An age-matched control group of three hundred current or former smokers was also included. A questionnaire was used for demographic data collection, along with the Hospital Anxiety and Depression Scale (HADS) questionnaire for the evaluation of anxiety (HADS-A) and depressive (HADS-D) symptoms. Results: The COPD and non-COPD groups were similar in age, gender, and socioeconomic background. The majority of COPD patients were classified as Grade 1 or 2 and belonged to Group A or B according to the GOLD classification. Among the COPD patients, 19.6% had a score greater than 7 in the HADS-A subscale, 14% in the HADS-D subscale, and 10.8% in both, compared with 6%, 5%, and 5%, respectively, for the non-COPD individuals (*p* < 0.01). A regression analysis showed that the presence of at least one comorbidity (β = 0.43, *p* < 0.001) and the presence of at least one respiratory symptom (β = 0.49, *p* < 0.001) significantly predicted the total HADS score in the COPD group. Conclusions: The prevalence of depression and anxiety symptoms in early COPD patients was greater in comparison to non-COPD smokers. Implementing routine screening for mood disorders using the HADS in mild to moderate COPD outpatients may improve overall disease management and patients’ quality of life.

## 1. Introduction

Despite being the third leading cause of death worldwide over the last several decades, COPD often remains underdiagnosed and undertreated, especially in terms of comorbidities, such as mood disorders [1,2]. Furthermore, even though anxiety and depression are known to be highly prevalent in people with chronic pulmonary diseases, COPD patients often do not receive sufficient care [3,4]. It is important for those who suffer concurrently from COPD and mood disorders to be treated for both to optimize their health-related quality of life, enhance their compliance with pulmonary rehabilitation programs, and improve their prognosis [5,6]. Since primary care professionals possess an essential role in preventive medicine and the management of a large number of COPD patients, they are tasked with the early detection of anxiety and depression symptoms [7]. Currently, the diagnosis and treatment of depression remain very limited in primary care settings, especially in cases of moderate depression [8].

The question that arises is “at what stage of COPD should we start searching for mood disorders?”, as depression and anxiety in these patients are multifactorial [9,10]. It has been suggested that mood disorders in COPD are related to its chronic course, to the physical inactivity that accompanies severe stages of the disease, and to the need for long-term oxygen therapy [11]. However, this has been challenged as, unexpectedly, it was found that severe COPD patients had a similar high prevalence of anxiety and depression regardless of whether they received long-term oxygen therapy [12]. Wagena et al. did not find a significant increase in psychological complaints in severe COPD patients compared with mild to moderate cases [13]. A number of authors have claimed that there is a direct causal association between COPD and mood disorders in the context of chronic systemic inflammation, while others disagree with that etiological mechanism [14,15,16,17,18,19,20]. Specifically, in a study of 120 moderate COPD patients, a weak correlation between depression and systemic inflammation, specifically TNFa and CRP, was found [18], whereas a significant association was discovered between IL-6 levels and depressive symptoms in a study of 745 COPD patients classified as Grades 2 and 3 [16]. In another study, higher serum levels of GDF-15, a stress-responsive cytokine, showed interrelationships with a sedentary lifestyle and cognitive risk in COPD patients [17]. However, the investigators of the ECLIPSE study could not prove a statistically significant correlation between systemic inflammation—specifically serum levels of hsCRP, IL-6, and fibrinogen—and depressive symptoms in COPD patients [19]. Therefore, the possible pathophysiological mechanisms are still unknown, and no clear relationship between COPD phenotypes and mood disorders has been established. Studies that have demonstrated the presence of depression or anxiety in patients with mild and moderate disease, who do not experience the disabling symptoms of severe COPD, support the hypothesis that chronic inflammation plays a key role in these disorders. The detection and management of mood disorders in the early stages of COPD may be beneficial for patients’ health [21,22,23,24]. In an extensive review on the prevalence of depression and anxiety in COPD patients, Panagioti and co-workers included five studies with moderate and severe patients, eleven studies with moderate COPD patients with rather small sample sizes (only three of them had a sample size greater than 100), and only one study of 187 patients with mild to moderate COPD [24]. Though Xiao and colleagues included a large sample of 275 patients with mild disease in their cross-sectional study, they had no control group [25].

Our study aims to contribute to the relevant research by providing evidence of the presence of anxiety and depressive symptoms in COPD patients at early stages of the disease. We investigated the presence of mood disorders in early COPD without chronic complications using a relatively large sample compared to those used in other studies, as well as a control group of people with a similar smoking history.

## 2. Materials and Methods

### 2.1. Study Design

This was an observational, cross-sectional, questionnaire-based study among current and former smokers, with and without COPD, selected from the population that participated in a study on early COPD detection and smoking cessation by the 3rd Regional Health Care Authority of Central Macedonia, Greece, and Aristotle University of Thessaloniki, between 1 January 2012 and 31 December 2019. The study protocol has been approved by the Scientific and Medical Ethics Committee of “G. Papanikolaou” General Hospital in Thessaloniki.

### 2.2. Participants—Inclusion/Exclusion Criteria

**Scheme 1:**
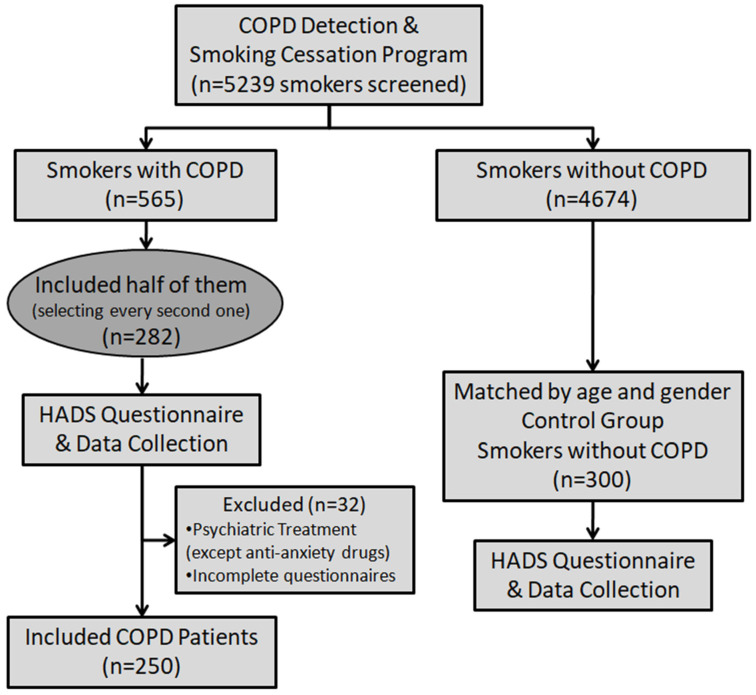
Patient flow diagram.

Participants were selected from a total population of 5239 subjects (>40 years old, current or former smokers, ≥10 pack-years), of whom 565 were diagnosed with COPD. Of these, 264 had a prior correct diagnosis of COPD, while the remaining were undiagnosed. We included half of the total COPD cases, starting with the first diagnosed patient and then selecting every second one thereafter. Therefore, the final COPD group consisted of 282 patients. Thirty-two patients were excluded from the analysis because they did not adequately fill out the study or HADS questionnaire, left unanswered questions, or were already receiving psychiatric treatment for a coexisting psychiatric disease. Mild anti-anxiety medications were excluded because they are commonly used by non-psychiatric patients. An age- and gender-matched control group of 300 subjects without COPD was also included (Scheme 1).

### 2.3. Data Collection

Demographic, socioeconomic, and clinical data related to COPD and its comorbidities were collected using a questionnaire designed by a group of experienced pulmonologists and administered at the Primary Health Care Center of Thessaloniki, Greece. The Greek version of the Hospital Anxiety and Depression Scale (HADS) questionnaire was self-administered by participants in a quiet outpatient setting, with trained staff available for assistance, to evaluate the presence of anxiety and depressive symptoms [26]. All participants successfully completed pulmonary function tests and answered all the questionnaire items.

### 2.4. Measurements

**Figure 1:**
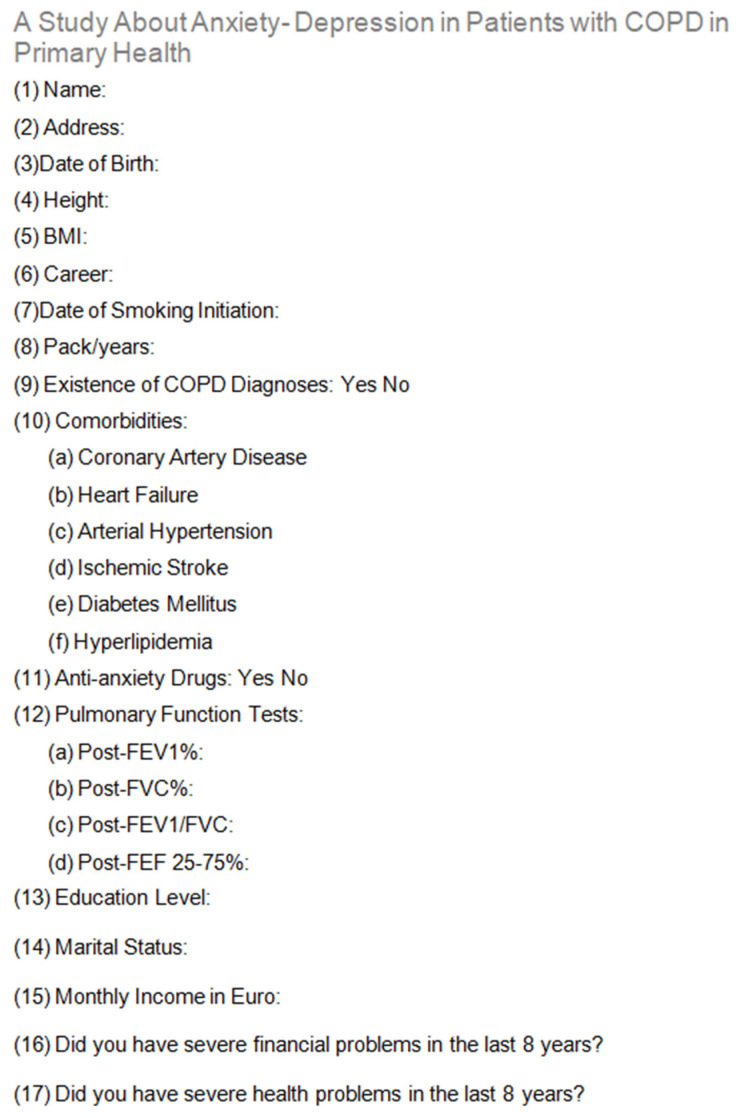
The questionnaire used in the present study for data collection.

The questionnaire used in the present study composed of 18 questions, of which 12 were open-ended regarding the patients’ demographics, 4 were yes/no close-ended questions about their COPD history and their experience of recent medical and financial problems, and 2 were multiple choice questions about their approximate monthly income and comorbidities (Figure 1).

The HADS is a well-known psychometric instrument used in various studies as a screening tool for depression and anxiety in COPD patients [27,28]. Although a psychiatric evaluation is the most reliable method of diagnosing mood disorders, and some question its accuracy for depression identification, the HADS has been proven to be a useful screening tool in the outpatient assessment by non-psychiatrists [29,30,31]. The HADS is a self-administered questionnaire composed of two subscales, one for anxiety (Hospital Anxiety and Depression Scale—A (HADS-A)), and one for depression (Hospital Anxiety and Depression Scale—D (HADS-D)). Each of them comprises seven items [32].

### 2.5. Variables and Definitions

#### 2.5.1. COPD

COPD diagnosis was confirmed through a pulmonary function test when the ratio of post-bronchodilator forced expiratory volume in one second to forced vital capacity (post-FEV_1_/FVC) was <0.70. The classification of COPD was based on an assessment of airflow limitation and clinical status according to the latest GOLD guidelines in 2023 [33]. To estimate the presence of respiratory symptoms, the modified Medical Research Council (mMRC) dyspnea scale and the COPD Assessment Test (CAT) were completed. The participants were mostly asymptomatic, as they were recruited in the context of a preventive program; however, many underestimated and ignored respiratory symptoms were revealed.

#### 2.5.2. Anxiety and Depressive Symptoms

Anxiety and depressive symptoms were identified through seven questions on each of the HADS-A and HADS-D subscales. The questions in the HADS-A are about whether the patient feels tense, experiences panic, has a sense that something awful is about to happen, or worries excessively. The questions in the HADS-D are about their interest in things that used to bring joy, their ability to laugh, and their experience of cheerful feelings. A score ranging from zero to seven is considered normal. A score above seven is considered abnormal [27,32].

### 2.6. Statistical Analysis

Statistical analysis was performed using SPSS version 23.0. Descriptive statistics were used to summarize demographic, clinical, and socioeconomic data. Continuous variables were expressed as the mean ± standard deviation (SD), and categorical variables were presented as absolute numbers and percentages. To compare continuous variables between the COPD and non-COPD groups, the Mann–Whitney U test was used as the data followed non-normal distribution (verified with the Shapiro–Wilk test). For categorical variables, the Chi-squared test was applied to assess associations between group membership and demographic or clinical factors. To examine the relationship between COPD classification (GOLD Grade and Group) and the presence of depressive and/or anxiety symptoms (defined as an HADS subscale score >7), subgroup analyses were performed within the COPD cohort using the Chi-squared and Fisher’s exact tests when the expected cell counts were below 5.

Multiple linear regression analyses were also performed to evaluate whether gender, body mass index (BMI), smoking history, educational level, marital status, income, comorbidities, and symptoms could predict the HADS score. Model assumptions for linear regression (linearity, independence, homoscedasticity, and normality of residuals) were verified graphically. Linearity was evaluated through scatter plots, the independence of errors was tested using the Durbin–Watson statistic, homoscedasticity was assessed via residual vs. fitted value plots, and the normality of residuals was verified through Q-Q plots.

## 3. Results

### 3.1. Participant Characteristics

#### 3.1.1. COPD Patients

The mean age of the COPD patients was 65 years; 70.8% were men, their mean BMI was 27.55 kg/m^2^, and they had a smoking history of 44 pack-years. The majority were classified as Grade 1 or 2 and belonged to GOLD Groups A and B. The mean values of the post-bronchodilation pulmonary function test measurements were as follows: FEV_1_: 74% pred., FVC: 92% pred., FEV_1_/FVC: 65.1, and FEF_25–75_: 41% pred. The most common comorbidities were coronary heart disease, arterial hypertension, and hyperlipidemia. Most participants had a secondary education, two children, an income of less than EUR 500, severe financial and health problems, and were married (Table 1).

#### 3.1.2. Non-COPD Individuals

The participants without COPD had a mean age of 64.5 years; 69.7% were men, the mean BMI was 28.9 kg/m^2^, and they had a smoking history of 30 pack-years. They mostly had coronary heart disease and arterial hypertension, a secondary education, two children, an income of less than EUR 500, severe financial and health problems, and were married (Table 1).

### 3.2. Outcomes

**Figure 2:**
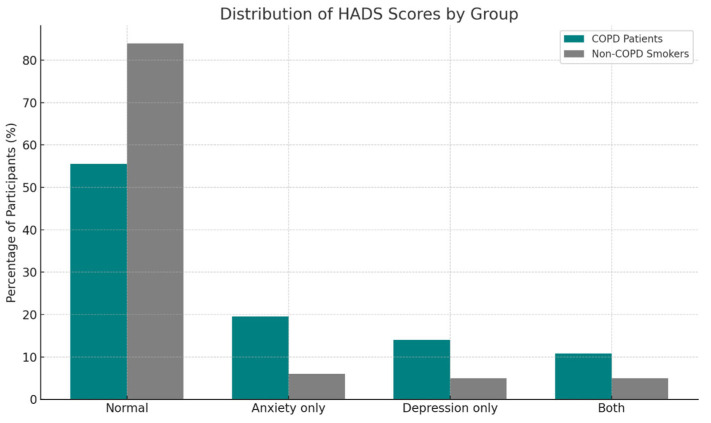
Distribution of HADS scores by group. Frequency of depression and anxiety symptoms (HADS score: normal ≤ 7; abnormal > 7).

A total of 139 COPD patients and 252 without COPD had a normal score in both HADS subscales. Among the COPD patients, 19.6% had a score greater than seven in the HADS-A subscale exclusively, 14% in the HADS-D subscale, and 10.8% in both. Regarding the control group, the percentages were 6%, 5%, and 5%, respectively (Table 2). Differences in both the total HADS score and the specific subgroup scores were statistically significant between the two groups (Figure 2).

We conducted a further subgroup analysis of the COPD subjects to compare those with a normal score less than seven in the HADS questionnaire and those with a score greater than seven. No statistically significant differences regarding demographic features and pulmonary function tests were found between the normal group and the depression and/or anxiety group. Subjects with more severe COPD (GOLD Groups B and E) were more likely to suffer from depression and/or anxiety (*p* < 0.05 and <0.001, respectively). The results of Fisher’s exact test show that there was no significant relationship between the comorbidities under investigation and the score in HADS (Table 3).

A battery of multiple linear regression analyses was performed to determine which variables influenced the total HADS score in both groups, namely the COPD patients and smokers with normal pulmonary function tests. In the COPD group, the overall regression model was statistically significant (R^2^ = 0.43, F(12, 237) = 15.41, *p* < 0.001), indicating that approximately 43% of the variance in the total HADS scores could be explained by the included variables.

It was found that the presence of at least one comorbidity (β = 0.43, *p* < 0.001) and the presence of at least one respiratory symptom (β = 0.49, *p* < 0.001) significantly predicted the total HADS score. This was also true for the HADS-A and HADS-D subscales. The other variables (age, gender, BMI, smoking history, educational level, marital status, and income) did not independently predict the HADS scores in the COPD cohort (all *p* > 0.05). In contrast, the regression model for the non-COPD smoker group was not statistically significant (*p* > 0.05), and no individual predictors reached significance. Νο multicollinearity issues were identified (all variance inflation factors were <2), and no major outliers were detected (Table 4).

No significant correlation was found between smoking history (pack-years) and the total HADS score, HADS-A, or HADS-D in either group (COPD patients and smokers with normal function tests), *p* > 0.05.

ROC analysis was performed to evaluate the predictive ability of respiratory symptoms and the presence of comorbidities in identifying elevated HADS scores. The results show the following:

- Respiratory symptoms: AUC = 0.73 (95% CI: X–Y);
- At least one comorbidity: AUC = 0.66 (95% CI: X–Y).

**Figure 3:**
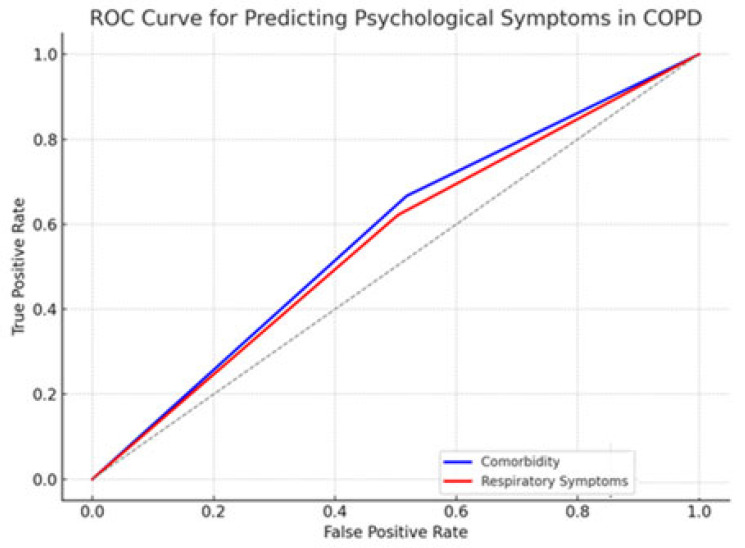
ROC Curve for Predicting Psychological Symptoms in COPD Patients.

It appears that the presence of respiratory symptoms was more strongly associated with the development of anxiety and/or depression symptoms in COPD patients (Figure 3).

## 4. Discussion

In comparing the COPD patients and non-COPD age-matched smokers, we observed an increased prevalence of both depression and anxiety symptoms in the COPD group (44.4% vs. 16%, *p* < 0.001). The presence of at least one comorbidity and of at least one respiratory symptom significantly predicted the total HADS score in the COPD patients. Nonetheless, no individual comorbidity independently predicted the HADS score. Regarding the non-COPD group’s characteristics, none of them predicted the HADS score.

The COPD patients with depression and/or anxiety symptoms (HADS questionnaire D and/or A > 7) and preserved pulmonary function (Grade 1: 53.1%) were much more symptomatic (Group B: 50.5%) compared to those without depression and/or anxiety (Group B: 38.1% and Grade 1: 27.3%, respectively). These results suggest that respiratory symptoms are more strongly associated with the presence of anxiety or depression symptoms in COPD patients, aligning with the study’s regression findings. These results support the utility of using symptom assessment for early screening of psychological distress in this population.

The majority of our COPD patients were classified as Grade 1 or 2 and in Group A or B according to the GOLD classification. They lacked many characteristics associated with depression and anxiety, such as low BMI, FEV_1_ < 50%, long-term oxygen therapy, and disability. Other potential confounders, namely low income, living alone, and dealing with severe health or financial problems, were also eliminated.

Our results for the prevalence of anxiety and depression in early COPD patients are consistent with those of other studies with similar populations [34,35]. Earlier studies have underestimated depression and anxiety in stage I (2006 GOLD report) or mild COPD, which may have been due to a different COPD classification method, an unequal distribution of patients among the stages, or not considering confounding factors. Our results are similar to those found in stage II COPD patients in the above-mentioned studies [36]. Overestimation of the prevalence of anxiety and depression among stage I/II COPD patients in other studies may be explained by a lower BMI, which is a risk factor for mood disorders and a sign of severe disease [37].

In our study, there were no significant differences regarding marital status, number of children, level of education, or income between the COPD and non-COPD participants. The COPD patients’ average BMI was significantly less than that of the non-COPD individuals, but it did not predict the HADS score in either group. Regarding the use of anti-anxiety drugs, we found that the COPD patients used them more frequently, which is consistent with the findings of recently published studies [37].

Cigarette smoking and nicotine dependence may explain the observed relationship between mood disorders and COPD, acting as confounding factors [38]. Though smoking history was expectantly heavier among the COPD compared to the non-COPD subjects (*p* < 0.01), no significant correlation was found between the number of pack-years and the HADS score in either group in our study. This indicates that the impact of smoking history is negligible.

In the subgroup analysis among the COPD patients, individuals with elevated HADS scores differed significantly in certain clinical and sociodemographic parameters compared to those with normal scores. Specifically, a higher proportion of symptomatic patients belonged to GOLD Group B rather than Group A despite having preserved lung function, suggesting that the presence of symptoms, rather than airflow limitation, may be more strongly associated with psychological distress in early COPD.

Although no statistically significant differences were found in the spirometric indices (FEV_1_, FVC, and FEF25–75%) between the groups, GOLD Grade 1 patients were paradoxically overrepresented in the anxiety/depression group. This finding supports the hypothesis that subjective symptom burden, rather than objective lung function impairment, may play a critical role in the development of mood disorders.

Furthermore, patients with primary education and those without children showed a higher prevalence of elevated HADS scores. This may reflect lower psychosocial support or health literacy, highlighting the need for targeted mental health assessment in vulnerable subpopulations.

The more frequent use of anti-anxiety medications in the affected subgroup (19 vs. 4 patients, *p* < 0.01) may represent either pre-existing conditions or unrecognized psychological burden and warrants further exploration.

Importantly, while comorbidities were prevalent in both subgroups, they were not independently associated with anxiety or depression symptoms, which is consistent with our regression findings. This suggests that the presence of symptoms and perceived burden of disease may be more salient than the number or type of comorbidities themselves.

Our study outcomes reinforce the clinical need to assess psychological wellbeing based not only on objective disease severity but also on the patient’s symptom experience and sociodemographic background. The limited number of participants in our study may have affected the results, although our sample size was similar to or greater than that of some other studies. A possible limitation is the rather small number of COPD patients with severe and very severe disease in our population, but this is unlikely to have an impact on the results in patients with mild and moderate COPD. Another limitation of this study is the possibility of residual confounding from unmeasured factors, such as undiagnosed psychiatric history, environmental exposures, or undetected cognitive decline. Additionally, the single time-point assessment of our patients prevented us from determining whether the onset of anxiety and depressive symptoms was established before or after COPD diagnosis.

Overall, we identified a higher prevalence of anxiety and depressive symptoms in COPD patients in the early stages compared to non-COPD individuals in Greece, while excluding contributing factors, such us smoking, BMI, and socioeconomic characteristics. Our results underscore the need for early identification and intervention of psychological symptoms in COPD, even at mild and moderate stages. The present study emphasizes the importance of routine screening for mood disorders using accessible tools, such as the HADS. Furthermore, early psychological assessment and subsequent management of depressive and anxiety symptoms may improve long-term outcomes, treatment adherence, and overall quality of life. Beyond standard medications, the most promising intervention to meet the challenges of managing depression in COPD patients is pulmonary rehabilitation [39].

## 5. Conclusions

There is a high prevalence of depression and anxiety in mild and moderate COPD patients compared to current or former smokers without COPD. Therefore, physicians in primary care should screen their patients for depression or anxiety in the early COPD stages, particularly at the time of initial diagnosis, in annual reviews, or in preventative healthcare programs. The detection and management of these disorders may have a positive impact on patients’ healthy quality of life, compliance to rehabilitation programs, and COPD prognosis. We also suggest the HADS as a practical and simple tool for evaluating the presence of depressive and anxiety symptoms in COPD outpatients. Secondly, these data may be a stimulant for future studies investigating a more direct etiological association between mood disorders and COPD.

## Figures and Tables

**Table 1:** Demographics, types, and frequencies of morbidities and socioeconomic characteristics of COPD patients and control group.

Characteristics	COPD Patients (n = 250)	Non-COPD Smokers (n = 300)	*p*-Value
Age (years)	65 ± 9.1	64.5 ± 9.28	NS
Gender (male%)	70.8	69.7	NS
BMI (kg/m^2^) *	27.55 ± 5.57	28.9 ± 4.87	<0.01
Smoking history (pack-years)	44 ± 30.49	30 ± 28.34	<0.01
At least one comorbidity (%)	58.4	67.7	NS
Coronary heart disease (%)	24.4	21	NS
Congestive heart failure (%)	3.2	0.7	<0.05
Arterial hypertension (%)	29.2	37.3	<0.05
Stroke (%)	3.2	4.7	NS
Diabetes mellitus (%)	11.6	10.3	NS
Hyperlipidemia (%)	24.8	31	NS
Anti-anxiety drug use (%)	9.2	4.3	<0.05
Education Level (%)			
Primary	33.2	29.3	NS
Secondary	48.4	50.3	NS
Higher	18.4	20.3	NS
Marital Status (%)			
Married	63.2	62.3	NS
Single	9.2	12	NS
Divorced	21.2	19.7	NS
Widowed	6.4	6	NS
Children(%)			
No children	19.2	22	NS
1	21.6	19	NS
2	40.4	42.3	NS
3	15.2	13.7	NS
4	2	1.3	NS
5	1.6	1.7	NS
Income < EUR 500 (%)	41.6	38	NS
Income EUR 500–1000 (%)	34	35	NS
Income > EUR 1000 (%)	24.4	27	NS
Financial problems (%)	68.8	65.3	NS
Health problems (%)	54.8	53.7	NS

* BMI: Body mass index.

**Table 2:** Anxiety and depression (HADS scores).

Characteristics	COPD Patients (n = 250)	Non-COPD Smokers (n = 300)	*p*-Value
Normal HADS Score (%)	55.6	84	<0.001
Borderline */Abnormal ** HADS (%)	44.4	16	<0.001
Elevated Depression (HADS-D > 7) (%)	14	5	<0.001
Elevated Anxiety (HADS-A > 7) (%)	19.6	6	<0.001
Elevated Depression and Anxiety (%)	10.8	5	<0.001

* Borderline scores: 8–10; ** abnormal scores: ≥11 in each HADS subscale.

**Table 3:** Subgroup analysis of the COPD subjects.

Parameters	Normal (n = 139)	Depression and/or Anxiety (HADS-D/A > 7) (n = 111)	*p*-Value
Age ^1^	64 ± 8.66	69 ± 9.57	NS
Gender (Males)	95 (68.3%)	82 (73.8%)	NS
BMI ^2^	28.07 ± 6.04	26.84 ± 4.76	NS
Pack-years	40 ± 27.03	46 ± 34.12	NS
COPD Classification Spirometry Stage			
Grade 1 ^3^	38	59	*p* < 0.01
Grade 2 ^4^	85	38	*p* < 0.01
Grade 3 ^5^	14	11	*p* < 0.01
Grade 4 ^6^	2	3	*p* < 0.01
COPD Classification Clinical Stage			
Group A ^7^	70	41	*p* < 0.05
Group B ^8^	53	56	*p* < 0.05
Group Ε ^9^	16	14	*p* < 0.05
Pulmonary Function Tests			
Post-FVC% ^10^	89 ± 18.16	97 ± 19.22	NS
Post-FEV_1_% ^11^	70 ± 15.9	77 ± 20.05	NS
Post-FEV_1_/FVC	64.6 ± 6.7	66 ± 7.78	NS
Post-FEF25–75% ^12^	41 ± 9.74	41 ± 11.46	NS
Patients With At Least One Comorbidity	74	72	NS
Coronary heart disease	30	31	NS
Congestive heart failure	2	6	NS
Arterial hypertension	43	30	NS
Ischemic stroke	3	5	NS
Diabetes	13	16	NS
Hyperlipidemia	39	23	NS
Anti-anxiety drugs	4	19	*p* < 0.01
Education Level			
Primary	28	55	*p* < 0.01
Secondary	79	42	*p* < 0.01
Higher	32	14	*p* < 0.01
Marital Status			
Married	88	70	NS
Children			
No children	31	17	*p* < 0.05
Income			
Less than EUR 500 ^13^	53	51	NS
Financial problems	88	84	*p* < 0.05
Health problems	70	67	NS

(1) Age in years; (2) BMI: body mass index in kg/m^2^; (3) Grade 1 (mild): FEV_1_ ≥ 80% predicted; (4) Grade 2 (moderate): 50% ≤ FEV_1_ < 80% predicted; (5) Grade 3 (severe): 30% ≤ FEV_1_ < 50% predicted; (6) Grade 4 (very severe): FEV_1_ < 30% predicted or FEV_1_ < 50% with respiratory failure; (7) Group A: mild symptoms assessed by mMRC dyspnea scale and CAT score, and fewer than 2 exacerbations not requiring hospitalization; (8) Group B: more symptoms assessed by mMRC and CAT score, and fewer than 2 exacerbations not requiring hospitalization; (9) Group E: frequent exacerbations regardless of the presence of symptoms; (10) post-FVC: post-bronchodilator forced vital capacity; (11) post-FEV_1_: post-bronchodilator forced expiratory volume in 1 s; (12) post-FEF25–75%: post-bronchodilator forced expiratory flow at 25–75% of forced vital capacity; (13) income in EUR.

**Table 4:** Regression analysis results.

Outcome	Predictors	Standardized β	*p*-Value
Total HADS Score (COPD)	≥1 Comorbidity	0.43	<0.001
Total HADS Score (COPD)	Presence of Respiratory Symptoms	0.49	<0.001
Total HADS Score (Non-COPD)	No significant predictors	-	>0.05

Model fit for COPD group: R^2^ = 0.43, F(12, 237) = 15.41, *p* < 0.001.

## Data Availability

Upon request, the corresponding author can provide further information.

## Abbreviations

The following abbreviations are used in this manuscript:

COPD	Chronic Obstructive Pulmonary Disease
HADS	Hospital Anxiety and Depression Scale
HAD-A	Hospital Anxiety and Depression—Anxiety Subscale
HAD-D	Hospital Anxiety and Depression—Depression Subscale
FEV_1_	Forced Expiratory Volume in the First Second
FVC	Forced Vital Capacity
FEF25–75%	Forced Expiratory Flow at 25–75 of the Vital Capacity
BMI	Body Mass Index
mMRC Dyspnea Scale	Modified Medical Research Council Dyspnea Scale
CAT	COPD Assessment Test
